# Opportunities for targeted therapies: trametinib as a therapeutic approach to canine oral squamous cell carcinomas

**DOI:** 10.21203/rs.3.rs-4289451/v1

**Published:** 2024-05-02

**Authors:** Santiago Peralta, William Katt, Cheryl Balkman, Scott Butler, Patrick Carney, Amy Todd-Donato, Matthew Drozd, Gerald Duhamel, Nadine Fiani, Jordan Ford, JENNIFER GRENIER, Jessica Hayward, Kristiina Heikinheimo, Kelly Hume, Elizabeth Moore, Rishi Puri, Skylar Sylvester, Sydney Warshaw, Suzin Webb, Andrew White, Alexandra Wright, Richard Cerione

**Affiliations:** Cornell University; Cornell University; Cornell University College of Veterinary Medicine; Cornell University; Cornell University; Cornell University; Cornell University; Cornell University; Cornell University; Prism Veterinary Dentistry; RNA Sequencing Core and Center for Reproductive Genomics. Cornell University, Ithaca, NY.; Cornell University; University of Turku; Cornell University; Cornell University; Cornell University; Cornell University; Prism Veterinary Dentistry; Cornell University; Cornell University; Cornell University; Cornell University

## Abstract

Oral tumors are relatively common in dogs, and canine oral squamous cell carcinoma (COSCC) is the most prevalent oral malignancy of epithelial origin. COSCC is locally aggressive with up to 20% of patients showing regional or distant metastasis at the time of diagnosis. The treatment of choice most typically involves wide surgical excision. Although long-term remission is possible, treatments are associated with significant morbidity and can negatively impact functionality and quality of life. OSCCs have significant upregulation of the RAS-RAF-MEK-MAPK signaling axis, and we had previously hypothesized that small-molecule inhibitors that target RAS signaling might effectively inhibit tumor growth and progression. Here, we demonstrate that the MEK inhibitor trametinib, an FDA-approved drug for human cancers, significantly blocks the growth of several COSCC cell lines established from current patient tumor samples. We further show clinical evidence that the drug is able to cause significant tumor regression in some patients with spontaneously occurring COSCC. Given the limited treatment options available and the high rate of owner rejection of these offered options, these findings provide new hope that more acceptable treatment options may soon enter the veterinary clinic.

## Introduction

Oral tumors account for approximately 6% of all neoplasms in dogs. Canine oral squamous cell carcinoma (COSCC) represents by far the most common oral malignancy of epithelial origin ^1^, exhibiting relatively high proliferation activity based on Ki67 immunostaining, particularly when compared to another common and locally invasive oral neoplasm of epithelial origin, canine acanthomatous ameloblastoma (CAA) ^2,3^. Importantly, up to 20% of dogs presenting with COSCC have regional or distant metastasis at the time of initial diagnosis, leading to difficulties in treatment ^1,4,5^. Locally, the primary tumor rapidly invades multiple anatomical planes including underlying osseous structures, resulting in severe pain, loss of function, and in some cases pathological fracture ^6^. In the absence of regional or distant metastasis, the treatment of choice for *COSCC* is *en bloc* excision of the affected area of the mandible or maxilla (i.e., mandibulectomy or maxillectomy) ^1,7,8^. Complete excision is associated with a high remission rate but is technically complex and expensive, and can result in significant disfigurement and dysfunction ^1,7,9,10^. Potential surgical sequelae include oronasal fistula, chronic ptyalism, protrusion of the tongue, and occlusal trauma ^1,7,11^. Conversely, incomplete excision often results in tumor recurrence and disease progression ^1^. Unsurprisingly, despite the high likelihood of cure, many owners choose not to pursue surgical treatment due to cost, morbidity, or both. Palliative courses of radiotherapy and chemotherapy have been described but are usually reserved for cases considered inoperable due to tumor size or the anatomical structures involved, for incompletely excised tumors, or for patients with regional or distant metastasis ^12–14^. Moreover, radiotherapy and chemotherapy are also associated with significant side effects and mixed outcomes ^15,16^. Thus, improved treatment options with reduced side effects are needed. Modern oncological targeted interventions based on small-molecule inhibitors have the potential to replace or complement traditional therapeutic options and help reduce patient morbidity while improving outcomes ^17,18^.

We have previously demonstrated that most canine oral tumors of epithelial origin exhibit significant activation of the RAS-RAF-MEK-MAPK (hereafter: RAS) pathway ^19–21^. This is similar to human head and neck cancers, which also frequently hyperactivate RAS pathway components ^22,23^. The RAS pathway is of major interest in oncology because it is mutated in a large percentage of cancers in humans and dogs ^24–27^. The RAS pathway has been recognized as a driver in many cancers, and hyperactivation of RAS and its signaling partners leads to increased and unrestricted tumor growth ^28–30^. Targeting the RAS pathway has therefore been a major objective globally. While RAS itself is traditionally difficult to inhibit directly, several of its downstream effectors have been targeted successfully with various inhibitors. One such target is MEK, a serine/threonine kinase that is activated by RAS and its partner RAF, and in turn activates MAPK and ERK in order to promote gene transcription and cell proliferation. Trametinib is a well-studied small-molecule inhibitor of MEK ^31^, has been approved for use in several indications in humans ^32,33^, and is being studied in combination with various other inhibitors to further improve its efficacy ^32,34–37^. Moreover, trametinib is specifically approved for use in human melanoma with a *BRAF* p.V600E or p.V600K mutation ^33^. Analogous *BRAF* p.V595E mutations are highly prevalent in COSCC, particularly in the papillary histological subtype of the tumor, which tends to proliferate very rapidly ^21^. Although less frequent, somatic *HRAS* p.Q61L mutations, which constitutively activate RAS and downstream signaling, have also been identified in COSCC ^2,19,20^. While trametinib has been shown to be effective in treating several canine cancers ^38–41^, this study is the first to demonstrate its efficacy in COSCC. Moreover, trametinib has been used “off-label” for canine histiocytic sarcoma ^42^. Taken together, these results suggest that trametinib may serve as an effective targeted therapy option for future management of neoplasms in dogs.

## Results

### Development of COSCC model systems

**Figure 1:**
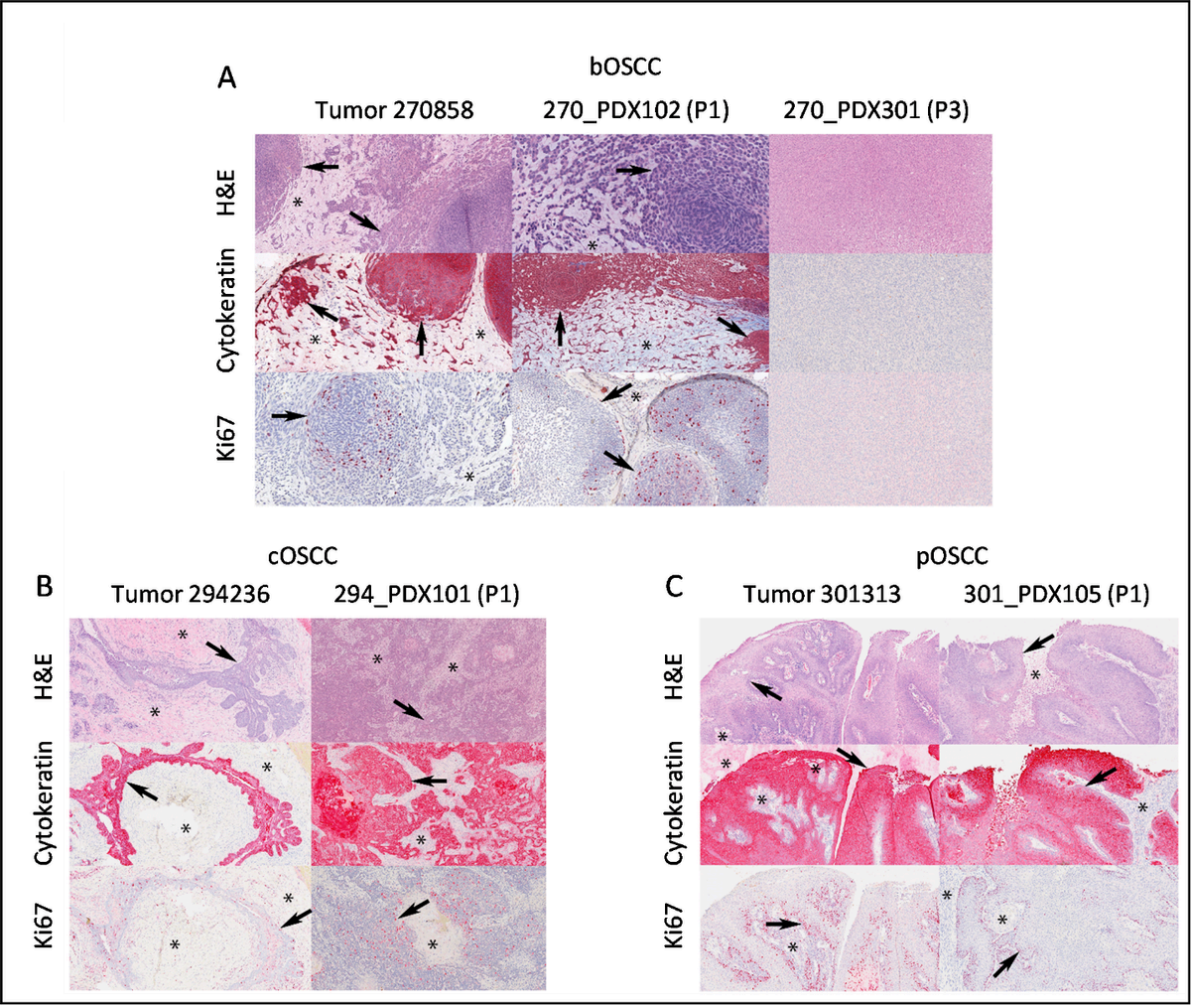
PDX Model Characterization. Representative 10X light photomicrograph images of formalin-fixed and paraffin-embedded samples taken from three primary COSCCs corresponding to the A) basaloid (bOSCC; tumor 270858,), B) conventional (cOSCC; tumor 294246) and C) papillary (pOSCC, tumor 301313) histological subtypes, and corresponding patient-derived xenograft (PDX) samples. Except for sample 270_PDX301 (P3) (panel A), which corresponded to a murine tumor that formed at the implantation site upon second passage, note the consistent neoplastic epithelial cell (arrows) invasion of the mesenchymal stroma (asterisks) in the primary tumor and PDX samples observed on hematoxylin and eosin (H&E) and pan-cytokeratin stains. Also, scattered Ki67 nuclear immunoreactivity of proliferating neoplastic epithelial cells (arrows) in the primary tumor samples is similar to the corresponding PDX samples.

Our overall goal was to develop model systems that would allow us to develop novel treatment strategies and translate them to clinical practice. We began by generating patient-derived xenograft (PDX) models in NOD scid gamma (NSG) mice. PDX models are widely considered to be among the most accurate models of a tumor, as they contain all of the diverse cell types a tumor is comprised by, and also maintain a tumor’s extracellular environment ^43–46^. We collected samples from dogs with spontaneously occurring OSCC tumors (Table 1). Minced tumor segments (average diameter 2–3 mm) were implanted into the left flank of NSG mice with within 6 hours of collection. The mice exhibited no signs of distress, and tumors generally became palpable in ~ 24–28 weeks. Tumors were then passaged into new mice at ~ 36–40 weeks. In each passage/generation, tumor sections were compared histologically to samples from the original tumor (Fig. 1). While one xenografted tumor (270858, Fig. 1A) appeared to have significantly altered phenotype by its second passage in mice, the other PDX models largely maintained the features of the parent tumor (Figs. 1B, 1C).

**Figure 2:**
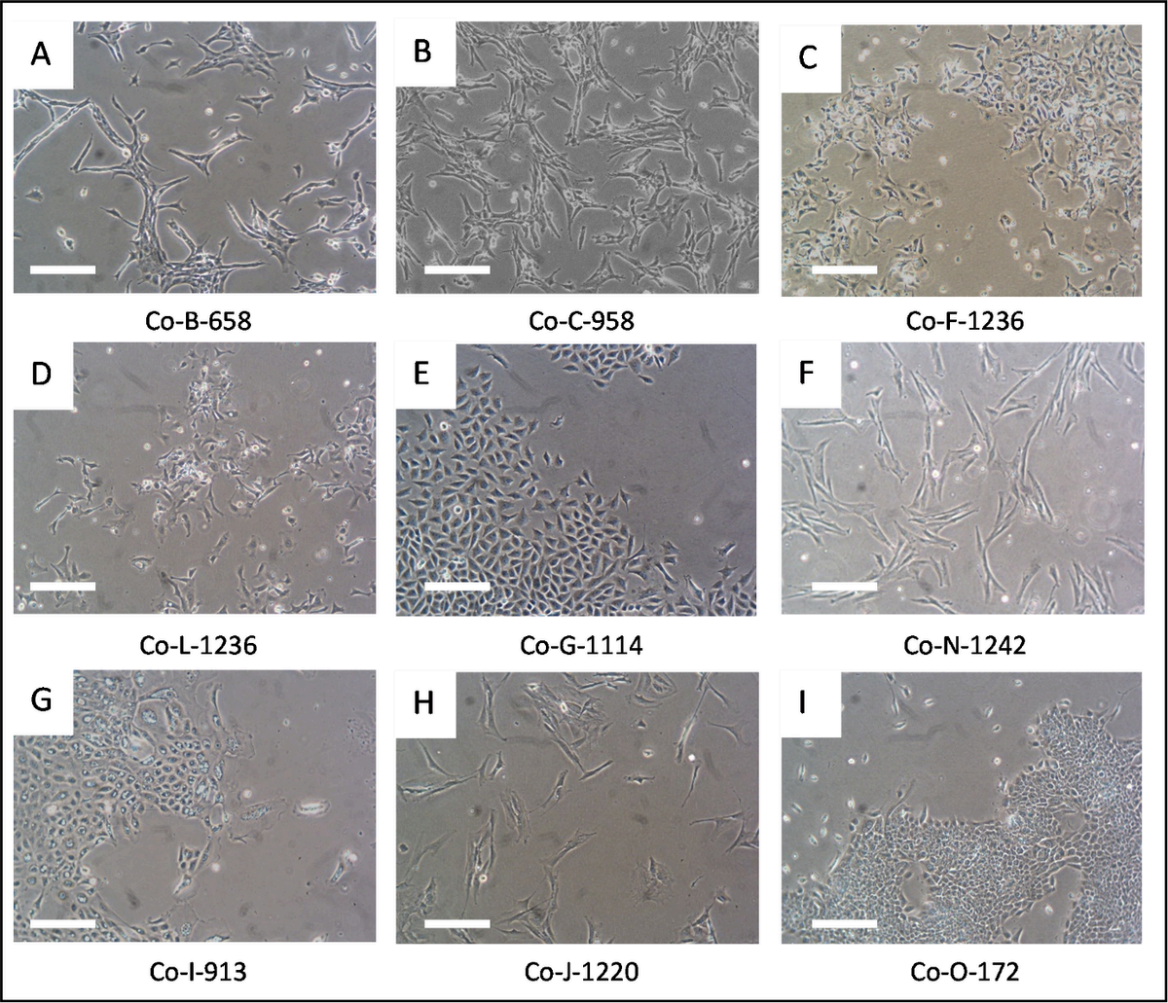
Micrographs of COSCC cell lines. Photographs of cells (A-I) were taken on a phase contrast microscope. Cells did not noticeably change morphology over ~10 passages/2–3 months. A different cell line is shown in each panel A-I, and the cell line name is labeled immediately underneath the photograph. Scale bar = 200 μM.

We next turned to developing stable 2D cell culture models of COSCC. Tumor samples were harvested from either our PDX-bearing mice (n = 5), or directly from patients (n = 2, Table 1). Two sequential passages of PDX tumor 270858 were used to generate cell lines Co-B-658 and Co-C-958. Two sequential passages of PDX tumor 294236 were used to generate cell lines Co-F-1236 and Co-L-1236. A single cell line was derived from each other tumor sample. The tumor tissue in each case was digested in collagenase II and trypsin, and epithelial cells were enriched gravimetrically ^47^. The cells were placed in tissue culture-treated plates, and the plastic-adherent cells were then cultured in RPMI 1640 medium supplemented with 10% fetal bovine serum (FBS). Figure 2 shows that the cells adopted a variety of different morphologies, but that different collections from genetically identical PDX models looked very similar, as expected.

COSCC is a disease of epithelial origin, and our goal was to enrich for epithelial cells while discarding infiltrating fibroblasts. However, differentiating between epithelial and fibroblastic cells in this situation is difficult. Gene set enrichment analysis previously demonstrated that COSCC tumors highly express genes supporting epithelial-to-mesenchymal transition ^19,22^, which can cause epithelial cells to adopt a fibroblast-like morphology and to express many of the same protein markers which might traditionally be used to identify fibroblasts (e.g. SNAIL or vimentin ^48–51^). Examination of our cell morphologies (Fig. 2) bears this out. Cell lines Co-G-1114, Co-I-913, and Co-O-172 (Figs. 2E, 2G, 2I) have block-like morphologies largely similar to other known epithelial cells. Co-L-1236 and Co-F-1236 (from genetically identical PDX model samples, Figs. 2C, 2D) are spindly, triangular cells appearing to be partway between epithelial and fibroblastic cell shapes and resemble some highly aggressive epithelial cell cancers such as the MDA-MB-231 human breast cancer cell line. Co-B-658 and Co-C-958 (from genetically identical PDX model samples, Figs. 2A, 2B), Co-N-1242 (Fig. 2F), and Co-J-1220 (Fig. 2H), meanwhile, are long, thin cells which look predominantly fibroblastic. Thus, more advanced characterization techniques than morphology analysis were required to help define these cell culture models.

### Model system characterization

**Figure 3:**
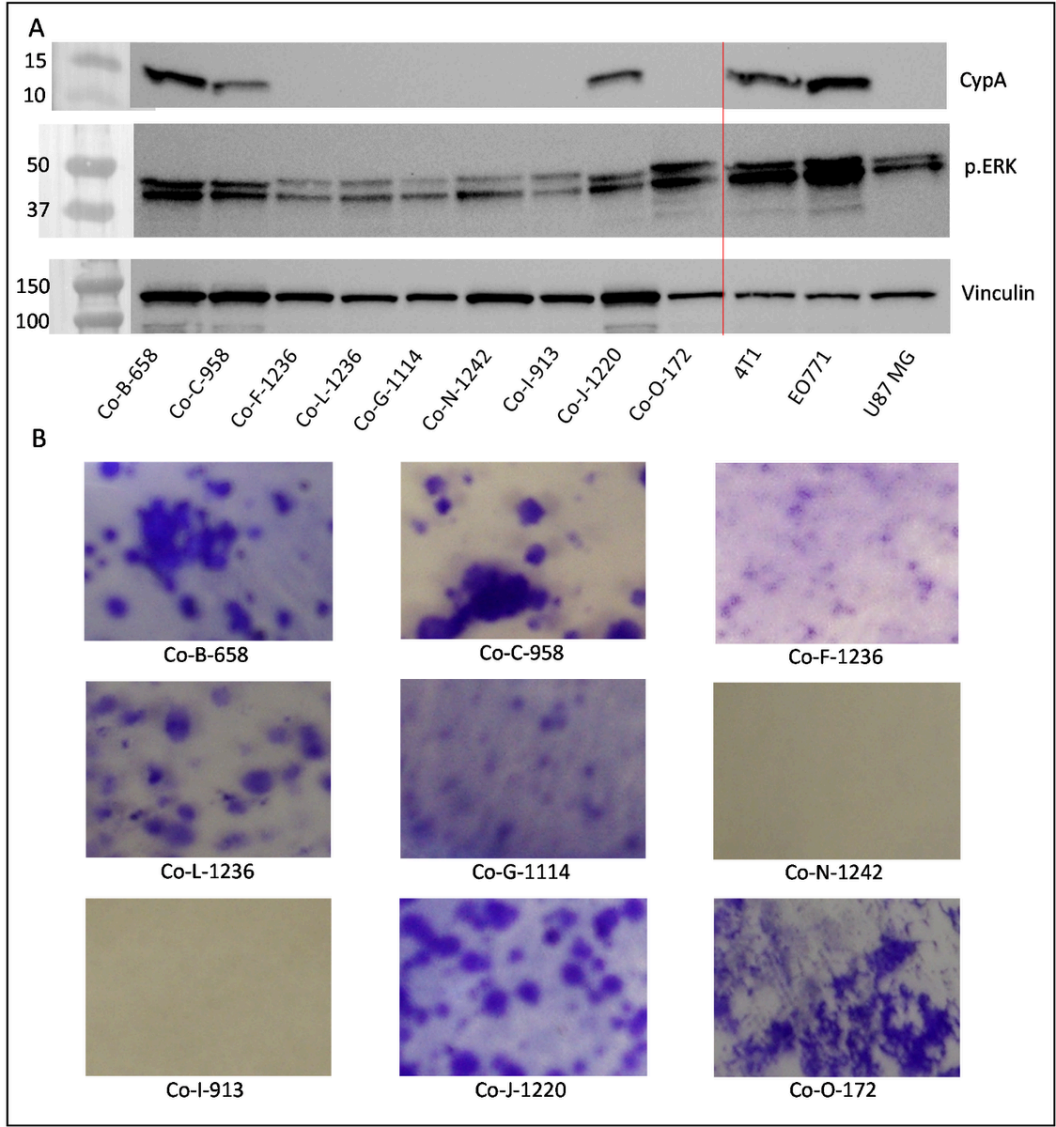
Characterization of COSCC cell lines. A) Cell lysates were resolved via SDS-Page, and blotted for murine cyclophilin A (CypA), phospho-ERK (p.ERK), or vinculin (loading control). 4T1 and E0741 are murine breast cancer cell lines, which show a signal for the protein, while U87 is a human glioblastoma cell line which is negative for the protein. Three of the novel cell lines show signal for murine protein. All of the novel cell lines have some phospho-ERK signal, suggesting intact RAS signaling. B) For each cell line, 1000 cells were plated into a 10 cm plate, and allowed to grow for 10–20 days. The plates were stained with crystal violet to visualize colony formation.

As we turned our attention to characterization of each model system, our major concern was determining whether each model was canine or murine in origin. We began by conducting Western blots for each cell line against a cyclophilin A (CyPA, an abundant and ubiquitous cytosolic protein) antibody known to bind murine protein specifically. This antibody had previously been demonstrated to selectively stain murine cells which invaded xenograft models of human cells in mice ^52^. The canine CyPA has an even lower sequence identity to murine CyPA than does the human protein (**Supplemental Figure S1**). Figure 3A shows that of the cell lines we isolated, three (Co-B-658, Co-C-958, and Co-J-1220) had detectable mouse protein, while the others did not. Two murine cancers (4T1 and EO771 breast cancer cells), and one human cancer (U87 MG glioma cells), are included on the blot for comparison. We had previously found that canine oral cancers have broad upregulation of RAS-RAF-MEK-ERK signaling ^19–21^, and so examined ERK phosphorylation as a marker of RAS activation. While phospho-ERK staining was different in each cell line (Fig. 3A), it was detectable in all cells to a similar extent to U87 cells, which are generally considered to have activated RAS signaling ^53^, thus demonstrating that RAS signaling was intact in our cell lines.

Following this, we addressed the issue of whether cells were derived from healthy or tumor tissue. Each cell line was cultured for a minimum of 10 weeks/15–20 passages to ensure some degree of immortality, with several cell lines being cultured for at least 50 passages/6 months. As healthy epithelial cells tend to senesce within 3–4 passages ^54^, this demonstrated a significant degree of immortalization in our cells, strongly suggesting that they were derived from tumor tissue. Notably, while we were able to passage cell lines Co-I-913 and Co-N-1242 for the 10-week period, both proved difficult to culture, and required that relatively high confluence be maintained at all times or the cells ceased to divide. The other cell lines were generally split from 70–80% confluence 1:10, and regained confluence within 3–5 days.

We next examined the ability of the cells to evade contact inhibition of growth, as most (but not all) cancer cells are able to sustain growth even following contact inhibition. Cells were plated at low density and allowed to form colonies (Fig. 3B). Most of the cell lines formed dense colonies under these conditions, strongly supporting a cancer phenotype. Cell lines Co-I-913 and Co-N-1242 did not form detectable colonies, but this was unsurprising given the previous observation that they must be kept at relatively high density to proliferate.

At this point, we wanted a simple mechanism by which to positively identify our cells as being related to the original tumors from which they were derived, and we turned to genotype analysis. The CanineHD beadchip (Illumina Inc.) contains approximately 220,000 single nucleotide polymorphisms (SNPs) across the canine genome ^55^. We collected DNA from tumor samples, PDX-samples, and cell line samples, and submitted the DNA for genotyping. Cell lines Co-B-658, Co-C-958, and Co-J-1220 failed due to high levels of SNP missingness. This strongly suggests that they are not canine cells and are instead immortalized mouse cells. Interestingly, we examined three different generations of the PDX tumor (270858) that led to the Co-B-658 and Co-C-958 cell lines. The first generation of the tumor was genotyped successfully, but the second and third generations (from which the cell lines were isolated) also had a high rate of SNP missingness. Combined with our histological analysis, which suggested a dramatic shift in tumor morphology from the original PDX tumor to later passages, this strongly supports the hypothesis that a murine tumor was growing from the second passage onward. The tumor and PDX samples leading to the Co-J-1220 cell line (tumor 303520) did not fail, suggesting that cell isolation may simply have captured spontaneously immortalizing murine fibroblasts.

We proceeded to analyze the genotype data from the samples which were canine in nature. We began by pruning the dataset - we removed SNPs that were missing in some data, or which were highly correlated, leaving 13,178 SNPs in the dataset. The data for each canine tumor, PDX model (1–3 per tumor), and cell line (1–2 per tumor) were then compared by calculating ^π (pi-hat), an identity-by-descent metric which takes into account the probabilities that 0, 1 or 2 alleles are shared between two samples ^56^. ^π values are ~ 1 if two samples are genetically identical. Table 2 shows that most tumor, PDX, or cell-line pairs from the same sample had ^π values over 0.97, suggesting near identity with one another. A notable outlier is Tumor 301814, which showed ^π of 0.82 compared to the derived PDX-model, and 0.76 compared to derived cell line Co-G-1114. The PDX-model and cell line were more closely related to one another, with ^π of 0.94, which suggests that more significant genetic mutation might have occurred while establishing the PDX model than while establishing the cell culture model.

**Figure 4:**
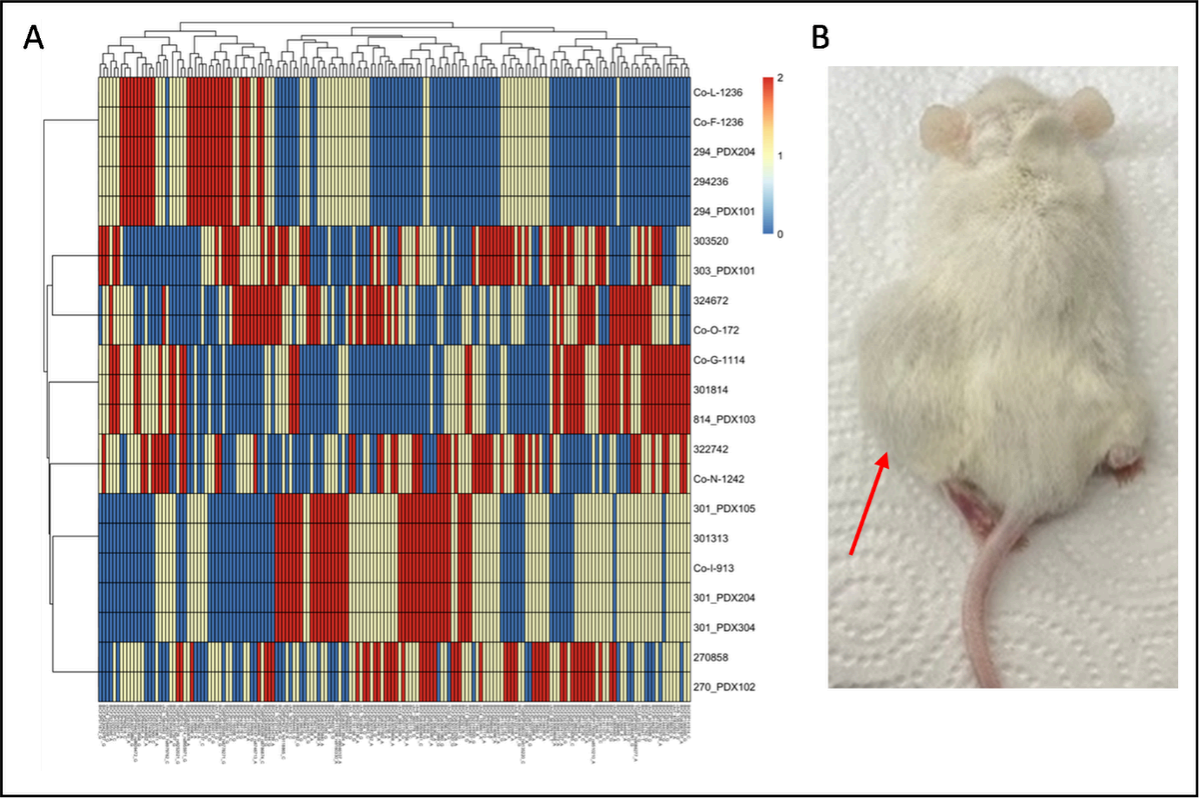
Further characterization of COSCC cell lines. A) Select SNPs were chosen from genotyping data in order to develop a ‘fingerprint’ for each COSCC cell line. Colors represent genotypes (0 = blue, 1 = yellow, 2 = red). B) Co-F-1236 cells were suspended in Matrigel and implanted into the left flank of NSG mice. The mice grew large tumors (indicated by red arrow) at the injection site.

Following this analysis, we selected 168 SNPs to form a ‘fingerprint’ for each cell line (Fig. 4A, **Supplemental Table S1**), which could be used as authentication in the future to determine the extent of genetic drift due to passaging, or the presence of contaminating cells. Pairwise identity by descent (IBD) measures using the 168 fingerprint SNPs calculated between samples in the present study and previously genotyped dogs (n = 880) shows that no other sample is closely related (all ^π values < 0.25) (**Supplemental Table S2**). Of note, cell line Co-L-1236 was genotyped twice: at passage 14 and at passage 42, and these had a pairwise ^π value of 0.979 using 13,178 SNPs and were identical using our 168 SNP fingerprint. As a final test, we wanted to determine if our cell culture models could reconstitute tumors in mice. NSG mice were implanted with either the Co-F-1236 or Co-G-1114 cell lines (2 × 10^6^ cells suspended in Matrigel) and tumors were allowed to grow. Growth for either line was initially slow, with Co-F-1236 taking ~ 4 months and Co-G-1114 taking ~ 12 months to form palpable tumors. However, when we transplanted a Co-F-1236 tumor into new mice as though it were a PDX model, we found that the host-adapted tumor grew rapidly, reaching a size mandating euthanasia (~ 2000 mm^3^) in ~ 2–3 months (Fig. 4B).

### New approaches to inhibiting COSCC growth

**Figure 5:**
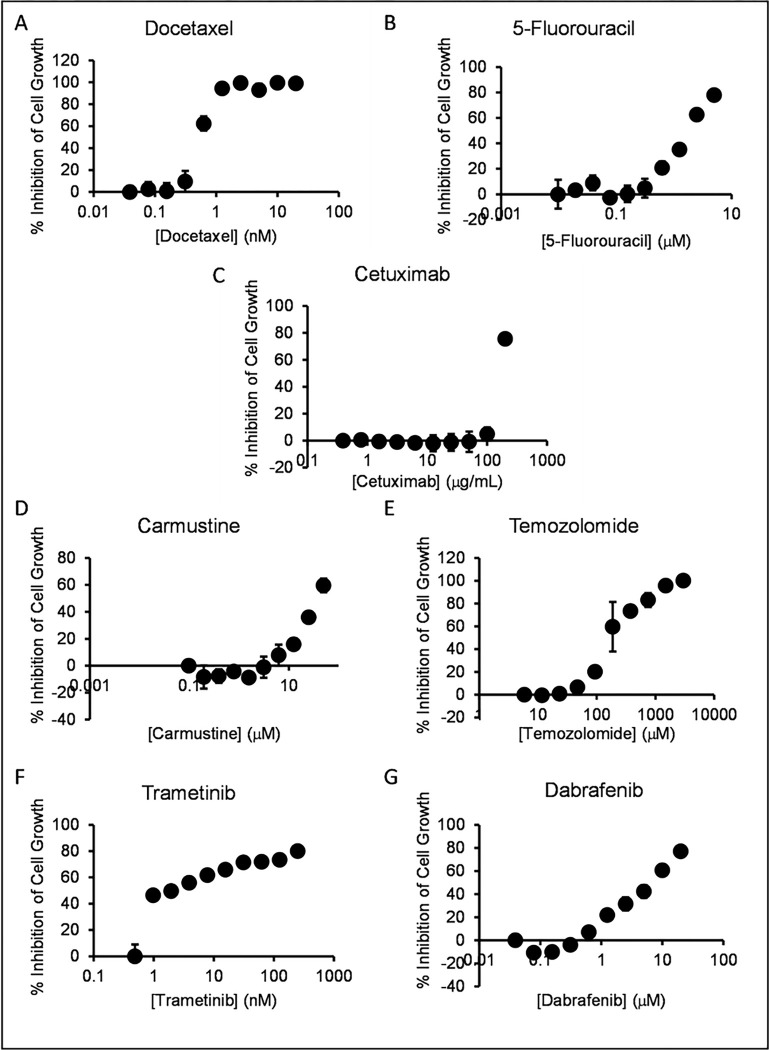
Dose curves for assorted drugs vs. COSCC cell line Co-F-1236. Cells were plated at low density (2,000 cells per well) in 96 well plates and treated for 6 days with the indicated amounts of each inhibitor (A) docetaxel, B) 5-fluorouracil, C) cetuximab, D) carmustine, E) temozolomide, F) trametinib, G) dabrafenib). The cells were then treated with PrestoBlue reagent, and cell viability was determined. %Inhibition was determined by comparison to no-drug controls (representing max growth) and cell-free lanes (representing 100% cell death). Each data point represents the average measurement from three samples, and the error bars represent the standard deviation from the mean. The lowest-concentration data point for each graph is a no-drug control and is given a non-zero value to be visible on the semi-log plot.

**Figure 6:**
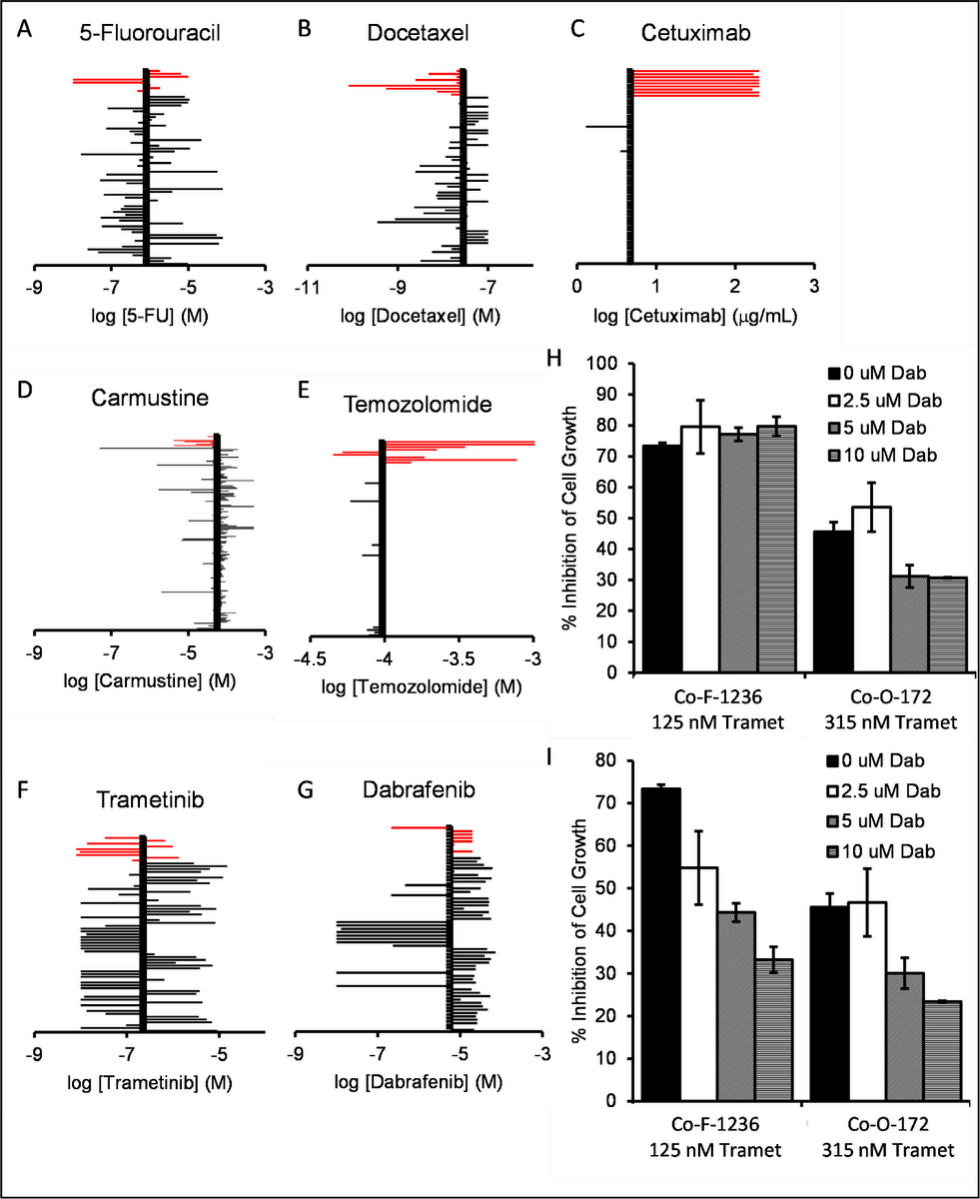
Sensitivity of COSCC to assorted drug approaches. A-G) The determined IC_50_ value for each drug (A) docetaxel, B) 5-fluorouracil, C) cetuximab, D) carmustine, E) temozolomide, F) trametinib, G) dabrafenib) for each COSCC cell line was plotted against data from the NCI-60. For each graph, the canine cell lines are highlighted as red bars, and the y-axis crosses the x-axis at the average IC_50_ value across all NCI-60 cell lines. Notably, the data for carmustine included an extended set of cell lines, so more bars are present than for the other drugs. H) Co-F-1236 or Co-O-172 cells were treated with trametinib (125 nM or 315 nM respectively) and the indicated amounts of dabrafenib (Dab: 0, 2.5, 5, or 10 μM) for 6 days, and %inhibition of cell growth was determined, as scaled to wells containing zero cells (100% inhibition) and wells containing cells not exposed to any drug (100% growth). Inhibition was only minimally additive for Co-F-1236 cells, while combination treatment actively reduced inhibition rate in Co-O-172 cells. I) As (H), except the data for each bar is scaled to wells containing dabrafenib alone (100% growth for that condition) to examine the effects of trametinib alone. Here, as the concentration of dabrafenib is increased, the effect due to trametinib appears to decrease.

With our model systems in hand, we turned our attention to testing novel therapeutic approaches. Since comparatively few approaches to COSCC have been demonstrated, we began by testing approaches that work against various cancers in humans. The current chemical standard of care for OSCC in people is based around three chemotherapeutics: docetaxel, cisplatin, and 5-fluorouracil (5-FU) ^18^. Cisplatin is comparatively difficult to handle due to chemical instability in solvating agents such as DMSO and the need to be prepared freshly before use, and so we began by studying docetaxel and 5-FU. Each cell line was dispensed at low density into 96 well plates and treated with different concentrations of each drug. Despite having been identified as murine cells, we conducted experiments on cell lines Co-B-658, Co-C-958, and Co-J-1220 as non-canine controls. Figure 5 shows the dose curves for each drug determined for cell line Co-F-1236, and IC_50_ data for all cell lines is compiled in **Supplemental Table S3.** These dose responses were compared to the average IC_50_ for either drug from the NCI-60, a set of 60 human cancer cell lines (Fig. 6). The doses for either drug were comparable to the NCI-60 averages, with no comprehensive difference between the canine cancer and murine cells. We proceeded to examine the EGF-receptor targeting antibody cetuximab, which has seen some use in human oral cancers ^57–59^. This showed very little potency in our COSCC cell lines compared to the NCI-60 average, although it is unclear if this is due to resistance to EGF-receptor inhibition, species specificity of the antibody, or simply due to the maximum concentration at which NCI-60 cell lines were tested impacting the statistical average efficacy. We then examined two other chemotherapeutic agents commonly used in some human cancers, carmustine and temozolomide. While the cell lines were generally sensitive to carmustine, they were comparatively resistant to temozolomide. And again, in either case, there was no clear difference between the sensitivity of canine and murine cell lines.

Each of the chemotherapeutics we examined is known for use-limiting side effects. We were curious if a more targeted agent might have similar effects in reducing COSCC growth. For this we chose trametinib, an FDA-approved drug which inhibits MEK1 and MEK2, downstream of RAS and RAF, and upstream of MAPK. Cells were exposed to trametinib (Fig. 5F), and the IC_50_ values compared to the average from the NCI-60. Interestingly, the cell lines were generally highly sensitive to trametinib when compared to other susceptible cancer cell lines (Fig. 6F). Notably, trametinib was the only tested inhibitor that had a strong effect on Co-I-913 cells, and it had the least effect on the three murine cell lines.

Trametinib is often given alongside the *BRAF* inhibitor dabrafenib, specifically in *BRAF* p.V600E mutant cancers ^31,32,37,60^. Several of our cell lines were derived from *BRAF* p.V595E papillary COSCC tumors, and we were curious if the two drugs could combine in these cell lines to provide a synergistic effect. We began by treating each cell line with dabrafenib alone and found mixed sensitivity to the drug as a monotherapy (Figs. 5G, 6G), with only one line (Co-O-172) being substantially more sensitive than the NCI-60 average, and four cell lines having very low sensitivity, with less than 50% inhibition of growth at 20 μM dabrafenib. We then treated two cell lines, the *BRAF*-mutant cell line Co-O-172 and the *BRAF*-wildtype cell line Co-F-1236, with trametinib and dabrafenib simultaneously. When compared to control DMSO-treated cells, the combination of trametinib and dabrafenib appeared to have a very mildly additive effect in Co-F-1236, but unexpectedly had a subtractive effect in Co-O-172 cells, where lesser inhibition of cell growth was observed with the combined drugs than with trametinib alone (Fig. 6H). However, when dabrafenib-treated cells were used as full-growth controls, and the effect of trametinib alone was calculated, it became apparent that the combination was antagonistic in both cell lines, with the effect due to trametinib decreasing as the concentration of dabrafenib increased (Fig. 6I).

### Trametinib treatment of COSCC in vivo

Trametinib as a single treatment had a significant effect on nearly all of our COSCC cell culture models, while combining it with dabrafenib resulted in only minimal improvement. Thus, we were interested to see if trametinib alone could reduce the growth rate of COSCC tumors in mice. Since our PDX-tumors were very slow growing, we chose to conduct testing on the aggressive and rapidly growing Co-F-1236 xenograft model. Cells (2 × 10^6^) were suspended in Matrigel, and then injected into the left flank of NSG mice. Once tumors became palpable, mice were randomly sorted into two cohorts (N = 6 per group). One cohort was treated with trametinib (0.5 mg/kg daily by oral gavage, 0.125 mg/mL drug stock concentration, **Figure S2**), and one was treated with an equivalent volume of vehicle alone (10% Cremophor EL, 10% PEG400, 80% PBS), similar to previously described protocols ^60,61^. The amount of drug administered was increased to 0.75 mg/kg on day 12, and mice were sacrificed due to tumor burden on day 25. Tumors were measured 3 times per week with calipers. Representative mice were subjected to MRI before drug treatment began, and immediately prior to sacrifice. Of note, a single mouse died the evening before mice were sacrificed, presumably due to aspiration of the liquid medication. It is included in all of our data due to the brief duration between its death and final tumor measurement.

**Figure 7:**
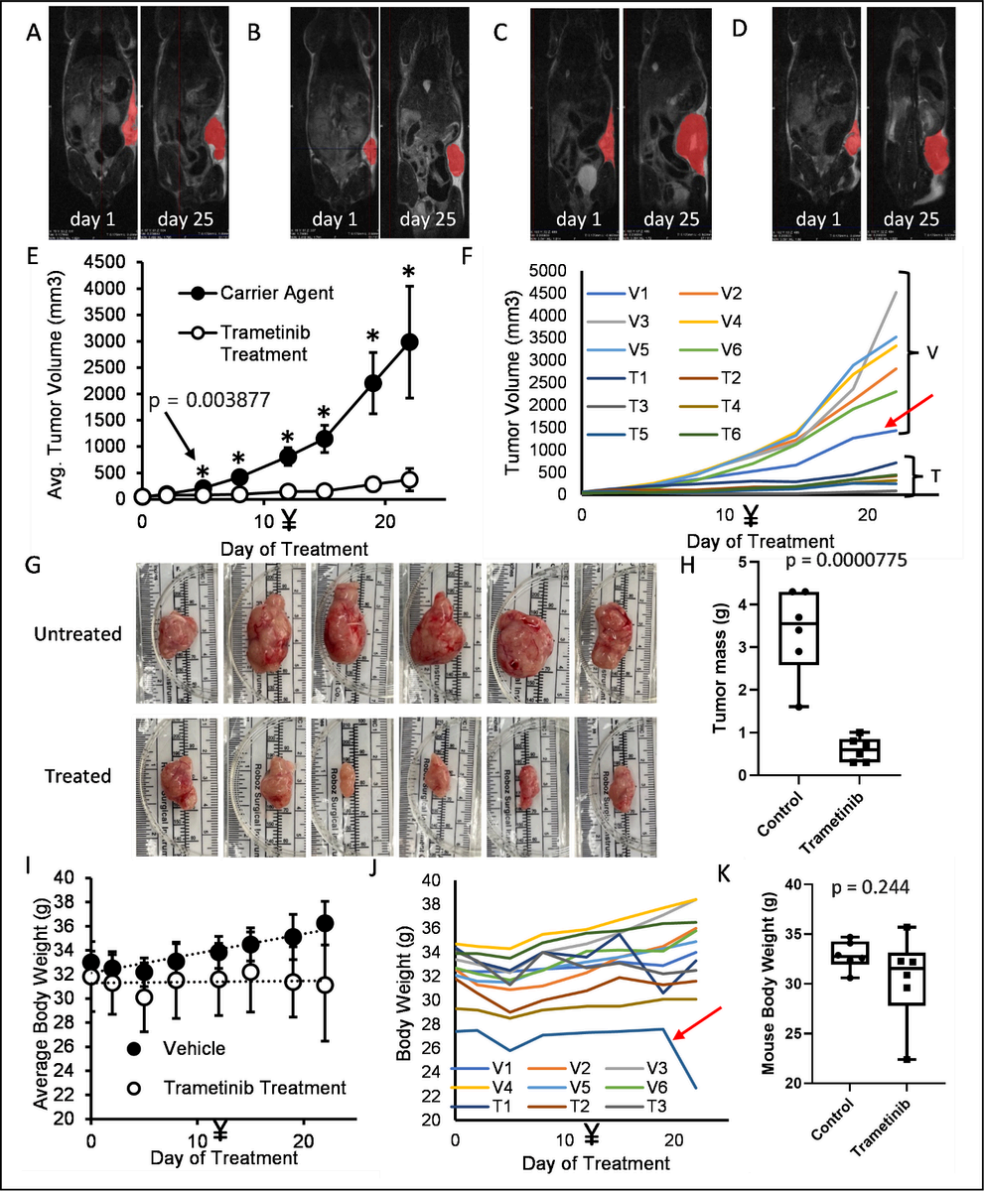
*In vivo* potency of trametinib against COSCC. A, B) MRI images of tumor-bearing mice before and after treatment with trametinib. Tumors are shown in red. C, D) MRI images of tumor-bearing mice before and after treatment with carrier control. Tumors are shown in red. E) Mouse tumors were measured (length and width) with calipers twice a week. Tumor volume was estimated as 0.5 * width (shorter measurement) * width * length (longer measurement). The drug treatment began at 0.5 mg/kg but was increased to 0.75 mg/kg on day 12 (marked with ¥). Datapoints represent the mean value from all six mice in each group, with error bars representing the standard deviation from the mean. The * represents data for which the p-value between treated and untreated samples is < 0.05. The exact p-value for the first statistically significant datapoint is shown. F) Individual growth curves for each mouse tumor. Curves are grouped into ‘vehicle’ (V) or ‘treated’ (T) groups. One mouse (V1, indicated with a red arrow) was treated only with carrier control, but had a comparatively slow growing tumor. G) Individual tumors removed from untreated and trametinib treated mice. Tumors were surgically removed from mice immediately following euthanasia, photographed and then weighed. Tumors from untreated mice are substantially larger than those from treated mice. H) The weights of tumors in (G) were determined. The heaviest trametinib-treated tumor was lighter than the lightest untreated tumor. I) The body weight of each mouse was determined immediately before drug was injected. Drug treatment had no noticeable impact on body weight, and untreated mice grew slightly heavier throughout the course of the experiment. J) Individual body weight plots for each mouse. One mouse in the drug treated group (T5, indicated with red arrow) was consistently lighter than all other mice, and died of treatment complications on the final day of the experiment, resulting in desiccation and significant body weight loss. K) The weight of each mouse’s tumor was subtracted from its total weight, and then the weights of the treated and untreated mice were compared. There is no statistical difference between their body weights on the final day of the experiment once tumor weights are controlled for.

MRI imaging conducted on representative mice before treatment, and immediately before sacrifice, show that trametinib treatment almost entirely halted tumor growth (single slice images are shown in Figs. 7A–7D). Measurement of tumor length and width with calipers and estimation of tumor volume (0.5 * width * width * length) showed a statistically significant difference between control and treated tumors by the 5th day of treatment, with drug-treated tumors showing little to no growth in that timeframe. However, even increasing the dose of drug on day 12 did not cause the tumors to shrink (Fig. 7E). Individual tumor sizes also clustered relatively well, with the exception of a single vehicle-control mouse with a slower growing tumor (Fig. 7F, red arrow). MRI and caliper measurements were mirrored by tumor appearance and weight (Figs. 7G, 7H). The mice did not show any signs of distress due to drug treatment, and no change in body weight was apparent (Figs. 7I–7K), with the exception of the one mouse which died prematurely (Fig. 7J, red arrow). Overall, these results show that trametinib was able to substantially block the growth of an aggressive COSCC tumor without having any acute, adverse effects on the mice hosting the tumor.

### Preliminary clinical results

**Figure 8:**
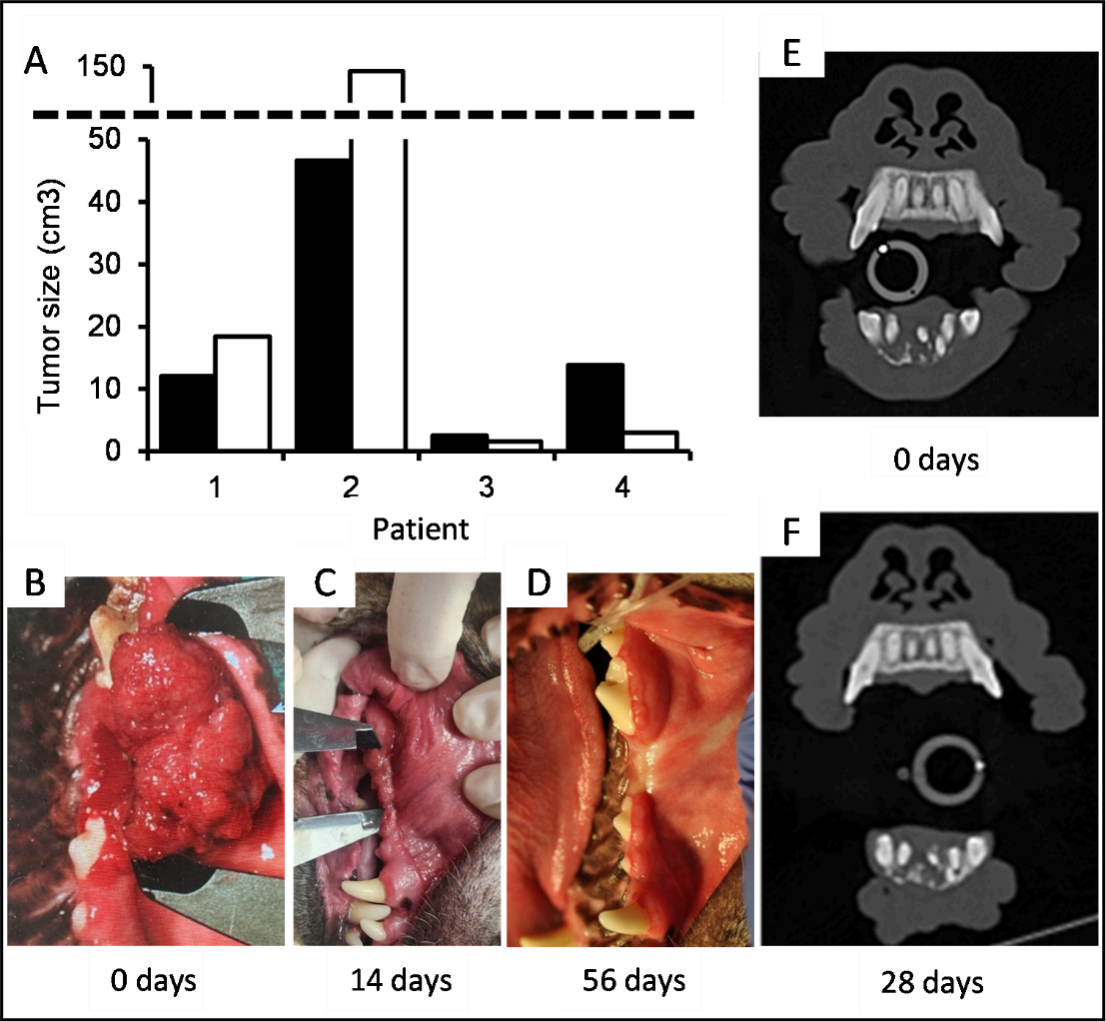
Preliminary clinical trial results. A) Chart showing initial tumor volumes when patients arrived for diagnosis (black bars), and at terminal point of trial (white bars). B) Photograph of patient 4 (see Supplemental Tables S4-S5 and Figures S3-S4) at the time of pre-enrollment, C) photograph of the same patient following 14 days of treatment with trametinib, D) photograph of the same patient at 56 days, upon exiting the trial. E) CT bone window slice of patient 3 showing osseous tumor invasion at initial diagnosis, and F) corresponding CT bone window slice of following 4 weeks of treatment with trametinib, exhibiting tumor size reduction and osseous regeneration.

Trametinib showed efficacy in our mouse CDX model and is an FDA-approved drug for which substantial safety testing has already been conducted ^32^. We were thus eager to determine if it could help dogs with spontaneously occurring oral tumors. While we cannot yet present the full results of our ongoing clinical trial, our preliminary results are encouraging and are presented here in full. For this, client-owned dogs diagnosed with COSCC that are older than 1 year, are not pregnant or lactating, have not previously undergone chemotherapy or radiation, and do not have signs of metastatic disease or other chronic disease, are enrolled. Qualifying dogs are prescribed 0.015 mg/kg or 0.020 mg/kg trametinib to be administered orally once per day. Dogs exit the study at day ~ 56, or if disease progression occurs based on biweekly medical examination including full tumor staging at week ~ 4. To date, we have treated four patients (**Supplemental Table S4 and Figure S3**), two of which exhibited a remarkable response to the drug, with tumors shrinking significantly (Fig. 8, **Supplemental Table S3 and Figure S4**), while the other two experienced tumor growth and exited the study. While we will need to recruit more dogs to make strong statistical arguments and explore differences across clinical and molecular phenotypes including mutational profiles, the fact that the two patients with COSCC responded well and without any observed toxicity is encouraging.

## Methods

Culture medium, FBS, and Tryp-LE were from Invitrogen. Collagenase type II and trypsin were from Worthington Biochemical Corporation. Trametinib was from Cayman Chemicals. All other chemicals were from Fisher unless otherwise noted. All animal procedures were approved by Cornell University’s Institutional Animal Care and Use Committee (IACUC), protocols 2005 – 0151 (clinical sample collection), 2017–0035 (experimental mouse work), and 2023–0034 (clinical trial).

### Tumor sample collection

After owners consented to sample collection, dogs were placed under general anesthesia. Two tumor samples were collected, with a 4 mm punch device for diagnostic use, and with a 2 mm punch device for deposit with the Cornell Veterinary Biobank (CVB). When the tumor was later surgically excised as part of the dog’s treatment, it was delivered to the Cornell University Progressive Development of Therapeutics (PATh) facility to generate PDX models. All procedures conducted on dogs were in accordance with accepted best practices and AVMA guidelines, and in accordance with approved Cornell University’s IACUC (protocol 2005 – 0151), and no extraordinary steps were conducted to obtain samples for use in these experiments.

### Histology

Tumor samples were embedded in formalin, and 4 μm slices were taken. These slices were mounted to charged slides, and then samples were processed with an automated IHC stainer largely as previously described ^62^. Paraffin was removed with Bond dewax solution (Leica), and then the samples were exposed to Bond epitope retrieval solution (Leica) for 30–40 minutes. Samples were then incubated with anti AE1/AE3 (DakoCytomation, #M3515, to detect cytokeratin ^2^) or anti-MIB-1 (DakoCytomation, #M7240, to detect Ki67), followed by alkaline phosphatase conjugated anti-mouse IgG (Leica, #PV6110) and RedDetection CM (Leica, #DS9390). Samples were alternately stained with hematoxylin and eosin stain. Slides were scanned with an Aperio CS2 ScanScope (Vista). All histological analyses were conducted by board-certified pathologists blinded to the condition being examined.

### Patient-derived xenograft development

NOD.Cg-Prkdc^scid^Il2rg^tm1Wjl^/SzJ (NSG) mice were bred in-house by the Cornell PATh facility, and were thus fully acclimated before experiments began. Tumor samples were collected from dogs as described above. Samples ~ 2–3 cm^3^ were placed in DMEM with penicillin and streptomycin for transport. Within 2–3 hours, the samples were minced into 2–3 mm^3^ pieces. Several samples were cryopreserved, and the remaining samples were divided evenly and implanted into five NSG mice (female, 6–8 weeks old). Mice were anesthetized with 2.5% isofluorane, and pieces were implanted into the left flank. Mice were administered analgesics as needed for pain and monitored carefully for two hours following surgery. Once tumors reached ~ 100 mm^3^, approximately 36 weeks after implantation, the mouse was sacrificed using carbon dioxide euthanasia (3.5 L/min), the tumor was harvested, minced into segments, and the segments were implanted into new mice (male or female as available, no more than 10 weeks old). Samples were also cryopreserved, and other samples were used to develop cell culture models as described below.

### Cell line development

Cell lines were isolated from tumors by adapting a procedure from a study on breast cancers ^47^. Briefly, tumor samples were collected from PDX-carrying mice, or directly from dogs. Tumors were soaked in 70% ethanol for ~ 30 seconds, and then minced with sterile blades. The tumors were washed twice with PBS to remove blood, and then transferred to a sterile conical tube. 5 mL of digestion buffer (2.5 mg/mL trypsin, 5 mg/mL albumin, 850 units/mL collagenase type II in PBS) was added to the tumor samples. The tube was capped and placed in a 37°C shaker and shaken at 550 RPM for 20 minutes. The mixture was then filtered through a cell strainer, and the tumor pieces captured by the strainer were transferred to a clean tube, suspended in 5 mL of fresh digestion buffer, and shaken for another 20 minutes. To this solution, 10 mL of wash medium (F12 medium supplemented with 5% FBS and 250 ng/mL gentamicin) was added. The tube was spun down (250 × g for 10 minutes), and 10 mL of medium (expected to contain primarily fibroblasts) was removed. The remaining 5 mL of medium, and tumor pieces, were expected to contain primarily epithelial cells.

Epithelial cells were collected via gravimetric enrichment. To the tube containing 5 mL of medium and tumor pieces, 10 mL of wash medium was added. The tube was inverted several times to suspend cells, and the cells were allowed to settle for 20 minutes. 10 mL of medium was removed and spun down to collect a cell pellet, which was suspended in RPMI-1640 supplemented with 10% FBS and dispensed into a single well of a 6-well plate. This ‘wash + collect + spin’ procedure was repeated 12 more times on the tube with tumor pieces, to create 13 culture samples. The tumor pieces were then suspended in RPMI 1640 + 10% FBS and dispensed into a well plate as well. Cells were kept highly confluent for 1–2 weeks, after which we began passaging them with greater dilution. In general, we found that each subsequent wash resulted in more homogeneous looking cells, but that cells in the high-wash wells were sometimes not confluent enough to grow successfully. We made use of the highest wash-number sample which showed robust growth to perform further experiments.

### Clonogenic colony formation assay:

Cells were grown to ~ 70% confluence, and then passaged using Tryp LE. Cells in suspension were counted, and 1000 cells were dispensed into a 10 cm dish. The cells were grown for 4 weeks, or until significant colonies were readily visible on inspection, whichever occurred first. The cells were then washed three times with PBS, fixed to the plates with 3.7% formaldehyde in PBS, and stained with 4% crystal violet in methanol for 5 minutes. The crystal violet was removed, and the cells were washed three times with water (5 minutes each wash) then one final water wash (2 hours). The plated colonies were then photographed.

#### Cell drug dose studies

Cells at ~ 70% confluence were washed with PBS and exposed to Tryp-LE solution for ~ 5 minutes. The cells were then suspended in RPMI-1640 supplemented with 10% FBS and transferred to a sterile conical tube. The cells were counted, then dispensed into 96-well plates at a density of 1000 cells per well. The cells were allowed to settle overnight, and the following day the medium was removed and replaced with medium containing the indicated DMSO-solvated drugs at assorted concentrations, or with DMSO alone as a control. The medium was again replaced on the fourth day of culture. On the sixth day of culture, the medium was removed, replaced with fresh medium, and cell viability was determined with Presto Blue reagent (Thermo). Viability was determined colorimetrically, following ~ 1–2 hours incubation with the reagent, on a Tecan Spark microplate reader. IC_50_ values were determined in GraphPad Prism using a two-parameter, variable slope logistic curve.

#### Western blotting:

Cells were grown to ~ 70% confluence, washed 3 times with PBS, and then lysed in lysis buffer (20 mM HEPES pH 7.6, 150 mM NaCl, 1 mM EDTA, and 1% NP-40). Protein concentration in the lysate was determined with BioRad Protein Assay Dye per the manufacturer’s instructions. 30 μg of protein in Laemmli buffer was then loaded onto 4–20% Tris-glycine gels and resolved via SDS-PAGE. Protein was transferred to PVDF membrane, and the membrane was blocked overnight in milk. The membrane was rinsed with TBST and stained for one hour with CypA antibody (1:1000 dilution in TBST, Cell Signaling #51418), phosphor-ERK antibody (1:2000 dilution, Cell Signaling #9106), or vinculin antibody (1:2000 dilution, Cell Signaling #13901). The membrane was washed 4X with TBST, and then exposed for two hours to HRP-linked anti-rabbit IgG (Cell Signaling #7074, 1:5000 dilution in TBST) or anti-mouse IgG (Cell Signaling #7076, 1:5000 dilution). The membrane was washed 4X with TBST, exposed to Western Lighting Plus Chemiluminescence reagent (Perkin Elmer), and imaged on a Bio-Rad ChemiDoc. Band density was quantitated using ImageJ ^63^.

#### Genotyping analysis:

DNA was collected from dog tumor, PDX tumor, and cell line samples using the Zymo DNA micro-prep kit per the manufacturer’s instructions. DNA was then submitted to Illumina for genotyping on the EMBARK version of the CanineHD 220k bead array. Quality control on the genotype data was performed in PLINK 1.9 (www.cog-genomics.org/plink/1.9/) ^56^. Samples with missingness values of > 3% were considered to have failed and were removed. For the remaining samples, SNPs with any missingness were removed, and then linked SNPs were removed, resulting in 13,178 informative SNPs being maintained. For each sample pair, the IBD was calculated using a metric called pi-hat (^π), which is a measure of the probability that the two samples share 0, 1 or 2 alleles. To generate a ‘fingerprint’ for each cell line, we selected 168 SNPs across the genome to differentiate the cell-lines from each other. Criteria for SNP selection included: no missingness, located > 10Mb apart, minor allele frequency (MAF) > 30% in the samples for the present study, and MAF > 30% in a group of 880 dogs genotyped on the same 220k array for other studies. Further manual selection was done to maximize genotype differences between the cell-lines. To show the relationship between each of the samples for these 168 SNPs, a heatmap was generated using the package pheatmap v 1.0.12 ^64^ in R ^65^. To determine the uniqueness of the SNPs chosen for fingerprinting, pairwise IBD was calculated using only the 168 SNPs for all samples in this study and the 880 dogs that have been previously genotyped.

#### In vivo drug study

Trametinib was dissolved in vehicle (10% Cremophor EL, 10% PEG400, 80% PBS). Co-F-1236 cells (2 × 10^6^) were suspended in Matrigel, and then injected into the left flank of male NSG mice (8–10 weeks old). Drug treatments began once the majority of mice had palpable tumors. Mice were randomly sorted into drug-treatment or vehicle-treatment groups. Mice were weighed daily to determine proper treatment volume. Six mice were treated with the indicated amount of trametinib daily by oral gavage, and six were treated with an equivalent volume of vehicle alone. Tumors were measured 3 times per week with calipers. Tumor volume was estimated as 0.5 * length * width * width. Representative mice were subjected to MRI before drug treatment began, and immediately prior to sacrifice. MRI images were analyzed using VivoQuant Imaging Software v3.5 by Invicro. At the experimental endpoint, mice were euthanized with carbon dioxide (3.5 L/min), then tumors were harvested, photographed, and immediately weighed before significant desiccation could occur. Data for tumor growth were analyzed for statistical significance using a two-tailed Student’s t-test.

### Ongoing clinical study

Client-owned dogs with a confirmed diagnosis of COSCC arising from an oral mucosal surface are enrolled in an 8-week study to evaluate the response and tolerability of trametinib. Eligibility requirements include completely staged patients that have no evidence of regional or distant metastasis, have not received previous treatment of the oral tumor (i.e., surgical excision, chemotherapy or radiation therapy), are considered suitable candidates to undergo general anesthesia, are not diagnosed with other debilitating or chronic systemic diseases, are not pregnant or lactating, and the owners can comply with the required follow up appointments during the study. All enrolled patients receive an oral dose of trametinib (0.015–0.02 mg/kg once daily) during the study. Response to therapy is monitored via a weekly phone call to document potential side effects observed at home by the owner, biweekly physical and oral examination including caliper measurements of visible oral mass and routine bloodwork (i.e., complete blood cell count and serum biochemistry panel), and full oral tumor staging under general anesthesia at days ~ 28 and ~ 56, including a contrast-enhanced (2ml/kg Iohexol 350mg iodine/ml) computed tomography (CT) head exam (16-slice Aquilion LB; Canon Medical Systems USA, Inc, Tustin, CA), diagnostic imaging of the abdominal cavity (CT or ultrasound) and thorax (CT or 3-view radiographs), and cytological or histological assessment of regional lymph nodes. The head CT exams are acquired in continuous helical axial slices, with slice thickness ranging from 0.5mm to 2.0 mm and reconstructed into volumes (effective slice thickness 0.3mm to 1.0 mm). The CT studies are reviewed by a board-certified veterinary radiologist as DICOM studies in the hospital’s Picture Archiving and Communication system [PACS] (Carestream VuePACS; Rochester, NY, USA) on medical diagnostic-quality monitors (Dell U3219Q; Dell Technologies, Round Rock, TX, USA). Tumor volume (cm^3^) is calculated as the sum of the hand-drawn cross-sectional area (cm^2^) of the abnormal [tumor] tissue on each axial slice, multiplied by the effective slice thickness. Tumor volume is determined on each head CT exam (timepoints: enrollment, d. 28, and d.56) and compared to document changes between each timepoint. All clinical procedures are done in accordance with a protocol (2023–0034) reviewed and approved Cornell University’s IACUC.

## Discussion

A major challenge in determining new treatments for veterinary patients is a relative lack of access to animal-derived cell culture model systems. In the case of COSCC, popular repositories such as the ATCC are extremely limited, with only 19 cell culture models available from *Canis familiaris* (domestic dog). Of these, only 1 cell line is derived from the head (the trachea, CF52.Tr cells), and it is not a cancer cell line. Of the dog cells, six are described as ‘normal’, eight as some form of cancer, and the rest lack a disease designation ^66^. This is a particular problem as drug responses are not identical even in samples of the same cancer type, and so ideally a large number of model systems will be used to determine if any given drug treatment is appropriate for a certain disease. To address this problem, we have developed several new model systems representing different COSCCs. Our PDX models largely replicate the phenology of their originating tumors, and our cell lines generally have morphology matching epithelial cells with high expression of epithelial-to-mesenchymal transition signals, which cause epithelial cells to develop fibroblast-like characteristics. Indeed, our cells do not look substantially different than some aggressive epithelial breast cancers, such as MDA-MB-231 cells ^67^, or ovarian epithelial cells ^68^.

We had developed multiple cell lines in order to get a relatively clear understanding of how well various drugs would work in COSCC. This approach proved to be important, as no drug had identical efficacy in all cell lines. We also noticed some differences in drug efficacy between each member of the two paired cell lines (Co-B-658 and Co-C-958, and Co-F-1236 and Co-L-1236), but these IC_50_ values were still generally similar when compared to other cell lines, suggesting that there were only minor differences between the cells from either isolation. That the differences between unique tumor-derived cell lines were generally larger than the differences between cell lines derived from the same tumor also suggests that approaches such as this might eventually be used for personalized medicine.

We investigated three different groups of drugs in this study: current standard of care agents for oral cancers in humans, other common chemotherapeutic agents, and agents which specifically target the RAS pathway. Encouragingly, our COSCC cell lines were generally highly sensitive to trametinib, an inhibitor of MEK1 and MEK2. Moreover, the COSCC cell lines were more sensitive than the three mouse cell lines we tested, and similarly more sensitive than the average human cancer cell line in the NCI-60. We did find that for most of the cell lines tested, the trametinib dose curve had a very broad slope, and that while a good effect could be obtained at relatively low concentrations of the drug, obtaining a more complete effect would require substantially more drug (Fig. 5F). That led us to attempt a combination treatment with dabrafenib, which is often co-administered with trametinib in humans, and has recently been tested in oral ameloblastomas ^37^. Unfortunately, the improvement in treatment efficacy in our COSCC cell lines was marginal at best (Figs. 6H, 6I). We suspect that this may reflect how both inhibitors target the same signaling pathway, and that more synergistic results may emerge in a whole tumor, where a heterogeneous cell population exists and amplifies the effects of single-drug resistance.

Happily however, the trametinib response alone was substantial in our 3D murine tumor graft models. We were also able to increase the dose from 0.5 mg/kg to 0.75 mg/kg in these *in vivo* experiments without the mice suffering any ill effect. While this increased dose also had no noticeably increased effect in reducing tumor growth, this does suggest a significant therapeutic window may be available for inhibiting COSCC growth without causing any negative side effects in the patient.

Trametinib is an FDA-approved drug in various indications and can be used off-label by veterinarians with little additional regulatory burden. The drug did not cause xenografted tumors to actually shrink, and instead only prevented growth. However, our xenograft model grew much faster than our PDX models do and was derived from a cell culture model that had already presumably selected for the fastest growing cells, suggesting that greater overall effects might be observed in a whole tumor. And indeed, our preliminary clinical trial results show two of four tumors shrinking after only a few weeks of trametinib treatment, with one large tumor almost entirely vanishing (Figs. 8B–8D). Although more data is necessary to assess which dogs with COSCC would be most likely to benefit from trametinib treatment, we remain excited by the results to date, and hope that continuation of our study will result in significant benefit to canine patients in the clinic.

## Conclusion

We have developed several new cell lines derived from COSCC tumors, either taken directly from dogs with spontaneously developing tumors or first passaged as PDX models in mice. These cell lines bear the phenotypic characteristics of cancer cells strongly expressing an epithelial-to-mesenchymal signal. While the cells are generally sensitive to several chemotherapeutic agents, they also show a strong sensitivity to trametinib, a MEK inhibitor used in some RAS-dependent cancers, even *in vivo*. Preliminary clinical results raise hope that the inhibitor may be of significant benefit to canine patients as well. Since targeted therapeutic agents generally have fewer side effects than traditional chemotherapeutics, we hope that these results will be a first step in deploying an effective, and well tolerated, treatment for COSCCs.

## Figures and Tables

**Table 1:** Characterization of tumors used to derive model systems.

Cell Line	Tumor	Age (yrs)	Sex	Breed	Tumor location	Diagnosis	*BRAF* p.V595E status	*BRAF* p.V595E status (blood)	Origin type
Co-B-658	270858	10	Female spayed	Dachshund	Rostral maxilla	bOSCC	WT	WT	PDX
Co-C-958	270858	10	Female spayed	Dachshund	Rostral maxilla	bOSCC	WT	WT	PDX
Co-F-1236	294236	7	Male castrated	Alaskan malamute	Caudal mandible	cOSCC	WT	WT	PDX
Co-L-1236	294236	7	Male castrated	Alaskan malamute	Caudal mandible	cOSCC	WT	WT	PDX
Co-G-1114	301814	11	Male castrated	Miniature poodle	Caudal mandible	cOSCC	WT	WT	PDX
Co-N-1242	322742	10	Female spayed	Yorkshire terrier	Caudal maxilla	pOSCC	UNK	UNK	Primary tumor
Co-I-913	301313	4	Female spayed	Labradoodle	Caudal maxilla	pOSCC	V595E	WT	PDX
Co-J-1220	303520	6	Female spayed	Labrador retriever	Caudal maxilla	pOSCC	V595E	WT	PDX
Co-O-172	324672	2	Female spayed	Labrador retriever	Rostral maxilla	pOSCC	V595E	WT	Primary tumor

*BRAF* status is reported as wild type (WT), unknown (UNK), or the specific mutant determined. Diagnosis is the histological subtype, either basaloid (b), conventional (c), or papillary (p) OSCC.

## Data Availability

Genotype data generated for this study and the 168 SNPs chosen for cell-line fingerprinting are publicly available as binary PLINK files at datadryad.org (doi:10.5061/dryad.zs7h44jh9).
